# Giant aneurysm of distal posterior inferior cerebellar artery: a case report and review of the literature

**DOI:** 10.1186/1752-1947-8-169

**Published:** 2014-05-29

**Authors:** Domenico Murrone, Danilo De Paulis, Massimo Gallieni, Mattia Del Maestro, Alessandro Ricci, Renato J Galzio

**Affiliations:** 1 Department of Neurosurgery, “San Salvatore” City Hospital, via Vetoio, Coppito, L’Aquila 67100, Italy; 2 Department of Life, Health and Environmental Sciences, University of L’Aquila, via Vetoio, L’Aquila 67100, Italy

**Keywords:** Clipping, Distal posteroinferior cerebellar artery, Giant aneurysm

## Abstract

**Introduction:**

Aneurysms in the vertebrobasilar system are rare and in the distal segment of the posterior inferior cerebellar artery they are even less frequent. Giant aneurysms are also rare in the posterior cranial fossa. Giant aneurysms of the distal posterior inferior cerebellar artery generally can have mainly compressive effects on the adjacent structures and they can be mistaken for tumors.

**Case presentation:**

We report the case of a 74-year-old Italian woman who presented with a complaint of dizziness. Her dizziness was found to be a result of aneurysmal dilatation arising from the distal segment of the right posterior inferior cerebellar artery. A mid-line suboccipital craniotomy was performed, and the aneurysm was clipped without post-operative deficits and with improvement in the patient’s dizziness. In our present report, we also review the literature and discuss our case with regard to the clinical and radiological features and surgical procedure performed.

**Conclusion:**

To the best of our knowledge, few cases of this type of aneurysm have been described in the literature. Our patient had a good outcome after surgical treatment.

## Introduction

Aneurysms of the vertebrobasilar system account for 5% to 10% of all intracranial aneurysms, and, at the level of the distal posterior inferior cerebellar artery, they are rare, comprising less than 0.5% to 3% [1-3]. They are referred to as giant aneurysms when they exceed 2.5cm in size. Giant aneurysms of the posterior inferior cerebellar artery (PICA) are very rarely found in the vertebrobasilar system. Half of all giant aneurysms are thrombosed, but complete obliteration of the aneurysmal sac is uncommon [4-6].

## Case presentation

**Figure 1:**
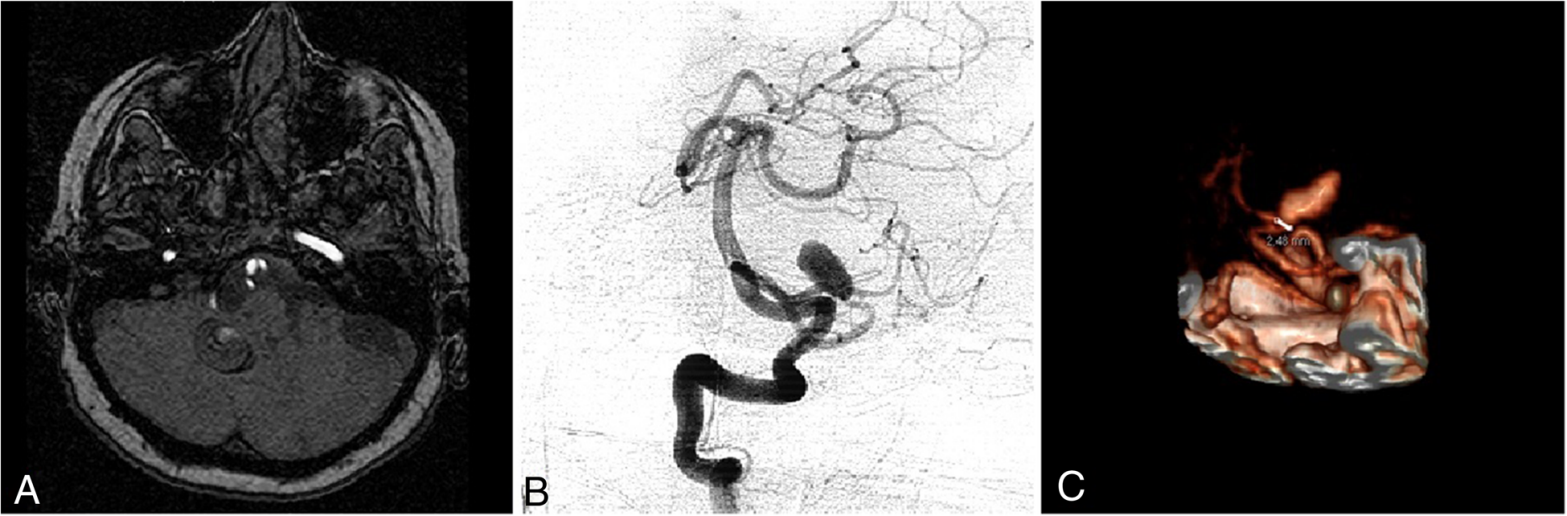
**Pre-operative magnetic resonance imaging studies. (A)** Post-contrast-enhanced T1-weighted magnetic resonance angiogram.** (B)** Digital subtraction angiography showing lateral view. **(C)** Three-dimensional reconstructed image showing the neck of the aneurysm.

**Figure 2:**
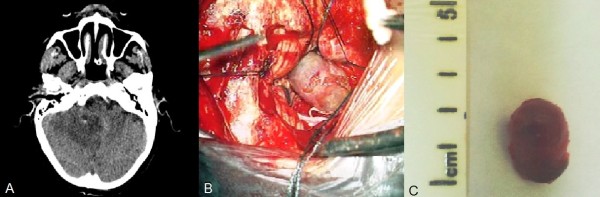
**Intra- and post-operative images of the aneurysm. (A)** Post-operative computed tomographic scan. **(B)** Intra-operative photograph of the aneurysm. **(C)** Photograph of the aneurysm after excision.

A 74-year-old Italian woman presented to our institution with complaints of severe headache and dizziness. Her neurological examination showed nuchal rigidity (Glasgow Coma Scale score 15 of 15 and Hunt and Hess grade 2) with gait ataxia. Magnetic resonance imaging (MRI) revealed a 2.8cm mass in the right cerebellar hemisphere with high signal intensity on T1-weighted images and low signal intensity on T2-weighted images, which were both associated with a peripheral signal void rim and not with peri-lesional edema. A magnetic resonance angiogram revealed aneurysmal dilatation arising from the distal segment of the right PICA and oriented medially with low signal intensity of flow only in part of the lumen and no signs of subarachnoid hemorrhage (SAH) (Figure 1). After endovascular coiling for vasospasm failed, we performed a mid-line suboccipital craniotomy with the patient under general anesthesia. Upon opening the cisterna magna, the cerebellar tonsils and the tonsillar loop of the PICA were exposed. The aneurysmal sac, originating from a loop at the telovelotonsillar segment, was identified and the proximal and distal portions of the parent artery were exposed and clipped for temporary occlusion with two YASARGIL clips (Aesculap, Center Valley, PA, USA). The aneurysm dome was isolated from the surrounding tissue, and, after an incision of the thick wall was made, an intra-aneurysmal thrombus was shaved with the ultrasonic aspirator. The neck of the aneurysm (2.48mm) was identified and clipped. Intra-operative Doppler ultrasonography was used to check preservation of blood flow in the distal PICA. The patient’s post-operative course was uneventful with neurological improvement, except for a transient admission to the intensive care unit (ICU). A post-operative computed tomography scan (CT) scan showed no hemorrhage or ischemia in the posterior fossa (Figure 2).

## Discussion

The PICA is the greatest of the branches of the vertebral artery and is the causative vessel for aneurysms in the posterior fossa, which cause cerebral infarction and cranial nerve compression in many patients. The PICA is formed by six segments and two loops. The telovelotonsillar and cortical segments are its distal parts. The bifurcation of the basilar artery and the origin of the PICA are the most common sites from which giant aneurysms arise in the vertebrobasilar system. Other locations within the posterior cranial fossa are much less common. Computed tomography reveals giant aneurysms as oval-shaped lesions with surrounding edema and, frequently, calcification. Contrast enhancement is strictly dependent on intra-aneurysmal thrombosis, but it may not be sufficient to confirm the diagnosis of the nature of the lesion, because it cannot differentiate these lesions from tumors. MRI can demonstrate a well-defined mass, with peripheral signal void rim and no contrast-enhancing components. Angiography is the most important diagnostic modality for revealing the true nature of the lesion, especially when there are no signs or symptoms of subarachnoid bleeding [5-7]. This exam should scan the presence of a contralateral PICA, the collateral circulatory pattern and the dominance, that are important radiological parameters, while deciding the choice of treatment. Multiple aneurysms can develop in the PICA [1]; they can be accompanied by arteriovenous malformations in rare cases; and they have low incidence of bleeding [2,3]. Giant intracranial aneurysms can be distinguished when completely thrombosed. They may escape angiographic identification when not thrombosed or partially thrombosed, which are the most common types [4-6]. The symptoms of ruptured PICA aneurysms are similar to those of subarachnoid hemorrhage Most patients who have unruptured PICA aneurysms present with compressive syndrome because these malformations usually appear clinically as space-occupying lesions and may occasionally be mistaken for tumors [4,7]. Early aneurysm treatment is necessary in patients presenting with SAH because rebleeding rates may be as high as 78%. The ideal treatment of a saccular lesion is clipping or endovascular obliteration of the aneurysm neck with preservation of the lumen. Giant aneurysms of the distal PICA are rare: to the best of our knowledge, only 14 cases of a total of 17 surgically treated patients have been reported [1-14]. Surgical treatment of these aneurysms has yielded good results [1,5,8,9,11,13], with clinical improvement, and only one patient died after surgery [14] (Table 1). The surgical approach for PICA aneurysms depends on the site of occurrence. For distal PICA aneurysms, suboccipital craniotomy is preferred. A different surgical option must be considered, however, when a dissection is performed, when the neck cannot be clipped without occlusion of the parent vessel or when small arteries arising from this segment pass through the brainstem. Trapping and excision of the aneurysm, as well as arterial reconstruction, performed by direct end-to-end anastomosis or insertion of an interposed arterial graft are possible treatment options [2,3,8,9,13,14]. An ultrasonic aspirator should be used for debulking of thrombosed or partially thrombosed aneurysms. Intra-operative fluoroangiography and Doppler ultrasonography can be very useful modalities for visualizing exclusion of the aneurysm from the circulation. Mortality can be related more to vasospasm as a result of subarachnoid hemorrhage rather than to technical aspects of surgery [1,2,4,6,15]. Endovascular treatment of distal PICA aneurysms with parent vessel occlusion may be an option. It has a complication rate as high as 13% due to the extremely variable and tortuous course of the PICA [7,9].

**Table 1:** **Reports of giant aneurysms of distal posterior inferior cerebellar artery**^**a**^

**Reports**	**Number of cases**	**Age/sex**	**Presenting symptoms**	**Treatments**	**Outcomes**
Hoeoek *et al*., 1963 [14]	1	50/F	PFS	Trapping	Death
Miller and Newton, 1978 [7]	1	61/F	PFS	NR	NR
Yoshii *et al*., 1979 [5]	1	72/F	PFS	Clipping	Good
Egashira *et al*., 1979 [6]	1	NR	PFS	Clipping	NR
Osenbach *et al*., 1986 [10]	1	NR	Ocular bobbing	Clipping	NR
Batjer, 1986 [9]	2	NR	Hemorrhage	Trapping in both cases	Good
Kusuno *et al*., 1986 [8]	3	37 to 66/2 M, 1 F	NR	Proximal ligation	Good
Dernbach *et al*., 1988 [1]	1	47/M	Asymptomatic	Clipping	Good
Osenbach, 1989 [11]	1	11 months/M	PFS	Clipping	Good
Richmond and Schmidt, 1993 [12]	1	67/M	FMS	Clipping	Good
Hamada *et al*., 1996 [13]	1	69/F	Ataxia	Clipping + anastomosis (SO)	Good
Drake and Peerless, 1997 [3]	1	70/F	NR	Trapping	Excellent
Lewis *et al*., 1997 [2]	1	52/M	Headache	Clipping + anastomosis (SO)	Good
Lim *et al*., 2008 [4]	1	64/F	Headache, hemiparesis	Clipping(SO)	Good
Present case	1	74/F	Headache	Clipping(SO)	Good

^a^FMS, Foramen magnum syndrome; NR, Not reported; PFS, Posterior fossa syndrome; SO, Suboccipital craniectomy.

## Conclusions

Giant aneurysms of the distal PICA are very rare and often present together with posterior fossa syndrome. On the basis of reports in the literature, we conclude that, for these aneurysms, the surgical option of direct clipping should be considered the first-line treatment. This option allows definitive obliteration of the aneurysm and possible removal of space-occupying lesions, especially in cases of thrombosed aneurysms. Endovascular treatment involves significant risks of neurological deficit due to the extreme variability and tortuosity of the PICA.

## Consent

Written informed consent was obtained from the patient for publication of this case report and any accompanying images. A copy of the written consent is available for review by the Editor-in-Chief of this journal.

## Abbreviations

CT: Computed tomography; MRI: Magnetic resonance imaging; PICA: Posterior inferior cerebellar artery; SAH: Subarachnoid hemorrhage.

## Competing interests

The authors declare that they have no competing interests.

## Authors’ contributions

All authors analyzed and interpreted the patient data and contributed to the writing of the manuscript. All authors read and approved the final manuscript.

## References

[B1] Dernbach PD Sila CA Little JR Giant and multiple aneurysms of the distal posterior inferior cerebellar artery Neurosurgery 1988 22 309 312 10.1227/00006123-198802000-00005 3352880

[B2] Lewis SB Chang DJ Peace DA Lafrentz PJ Day AL Distal posterior inferior cerebellar artery aneurysms: clinical features and management J Neurosurg 2002 97 756 766 10.3171/jns.2002.97.4.0756 12405360

[B3] Drake CG Peerless SJ Giant fusiform intracranial aneurysms: review of 120 patients treated surgically from 1965 to 1992 J Neurosurg 1997 87 141 162 10.3171/jns.1997.87.2.0141 9254076

[B4] Lim DH Jung S Jung TY Kim TS An unusual case of a thrombosed giant distal PICA aneurysm simulating a large cavernous angioma J Korean Neurosurg Soc 2008 43 155 158 10.3340/jkns.2008.43.3.155 19096624 PMC2588237

[B5] Yoshii Y Maki Y Egashira T Giant aneurysm of the distal portion of the posterior inferior cerebellar artery Eur Neurol 1979 18 382 386 10.1159/000115108 546660

[B6] Egashira T Yoshii Y Maki Y A case with giant aneurysm of distal portion of the posterior inferior cerebellar artery [in Japanese] No Shinkei Geka 1979 7 687 690 471179

[B7] Miller EM Newton TH Extra-axial posterior fossa lesions simulating intra-axial lesions on computed tomography Radiology 1978 127 675 679 663158 10.1148/127.3.675

[B8] Kusuno K Yoshida Y Shibata N Hayashi T Three cases of giant aneurysm of the distal posterior inferior cerebellar artery [in Japanese] No Shinkei Geka 1986 14 345 350 3703133

[B9] Batjer HH Multiple peripheral aneurysms of the posterior inferior cerebellar artery [Comment] Neurosurgery 1986 19 288 289 10.1227/00006123-198608000-00021 3748362

[B10] Osenbach RK Blumenkopf B McComb B Huggins MJ Ocular bobbing with ruptured giant distal posterior inferior cerebellar artery aneurysm Surg Neurol 1986 25 149 152 10.1016/0090-3019(86)90284-3 3941984

[B11] Osenbach RK Giant aneurysm of the distal posterior inferior cerebellar artery in an 11-month-old child presenting with obstructive hydrocephalus Pediatr Neurosci 1989 15 309 312 10.1159/000120488 2489589

[B12] Richmond BK Schmidt JH 3rd Giant posterior inferior cerebellar artery aneurysm associated with foramen magnum syndrome W V Med J 1993 89 494 495 8296475

[B13] Hamada J Nagahiro S Mimata C Kaku T Ushio Y Reconstruction of the posterior inferior cerebellar artery in the treatment of giant aneurysms: report of two cases J Neurosurg 1996 85 3 496 499 10.3171/jns.1996.85.3.0496 8751638

[B14] Hoeoek O Norlen G Guzman J Saccular aneurysms of the vertebral-basilar arterial system: a report of 28 cases Acta Neurol Scand 1963 39 271 304 14082563 10.1111/j.1600-0404.1963.tb05332.x

[B15] Spiriev T Kondoff S Schaller B Trigeminocardiac reflex during temporary clipping in aneurismal surgery: first description J Neurosurg Anesthesiol 2011 23 271 272 10.1097/ANA.0b013e3182204c2c 21654337

